# Optical Coherence Tomographic Features and Prognosis of Pneumatic Displacement for Submacular Hemorrhage

**DOI:** 10.1371/journal.pone.0168474

**Published:** 2016-12-19

**Authors:** Kunho Bae, Ga Eun Cho, Je Moon Yoon, Se Woong Kang

**Affiliations:** Department of Ophthalmology, Samsung Medical Center, Sungkyunkwan University School of Medicine, Seoul, Korea; Boston University School of Medicine, UNITED STATES

## Abstract

**Purpose:**

To identify prognostic factors, including optical coherence tomographic features, of visual outcome in exudative age-related macular degeneration with submacular hemorrhage treated with pneumatic displacement.

**Methods:**

This retrospective interventional case series included 37 eyes with exudative age-related macular degeneration and submacular hemorrhage, all of which underwent pneumatic displacement. The best-corrected visual acuity (BCVA) was measured at diagnosis and at 3 and 6 months after treatment. In addition to demographic and funduscopic parameters, tomographic features such as reflectance of the submacular hemorrhage were analyzed with regard to BCVA at 6 months.

**Results:**

After pneumatic displacement and a subsequent treatment such as laser or anti-vascular endothelial growth factor therapy, the BCVA at 3 and 6 months improved significantly (*P* < 0.001, respectively). Higher baseline BCVA (*P* < 0.001), shorter symptom duration (*P* = 0.007), and younger age (*P* = 0.014) were significant positive prognostic factors on regression analysis. Among optical coherence tomography characteristics, reflectance of the submacular hemorrhage, the shortest radius of the submacular hemorrhage centered on the fovea, and defects in the ellipsoid zone, and external limiting membrane affected the BCVA at 6 months (*P* < 0.05).

**Conclusion:**

A favorable visual outcome was demonstrated after initial pneumatic displacement and subsequent treatment for submacular hemorrhage. The submacular hemorrhages exhibiting lower reflectance on optical coherence tomography and a smaller shortest radius from the foveal center were found to be good candidates for pneumatic displacement.

## Introduction

Submacular hemorrhage can cause deterioration of visual acuity, and this occurs suddenly and often irreversibly in exudative age-related macular degeneration (AMD) and polypoidal choroidal vasculopathy (PCV) [1–3]. Various treatment choices, including vitrectomy and pneumatic displacement with or without tissue plasminogen activator, have been used to treat this condition [4–8]. Among these approaches, pneumatic displacement with intravitreal gas injection is relatively simple and less invasive compared to surgical drainage of the submacular hemorrhage [9]. The mechanism involves shifting the submacular hemorrhage from beneath the fovea while protecting the photoreceptor and retinal pigment epithelium (RPE) using the positive buoyancy of the gas. Previous studies have investigated prognostic factors of visual outcome in eyes with submacular hemorrhage after pneumatic displacement [4,10,11]. However, these studies were conducted before the introduction of anti-vascular endothelial growth factor (VEGF) and spectral-domain optical coherence tomography (OCT). In addition, we postulated that the liquid form of submacular hemorrhage is more advantageous compared to the coagulated, organized form in order to achieve successful displacement. However, the assessment of liquidity of submacular hemorrhage using reflectance measurement on OCT and its correlation with visual outcome has not been studied.

The purpose of this study was to elucidate visual outcomes in eyes with submacular hemorrhage initially treated with pneumatic displacement and to identify prognostic factors, including tomographic characteristics, of the visual outcome.

## Materials and Methods

This retrospective study was a single-center study that was conducted in accordance with the tenets of the Declaration of Helsinki. This study was approved by the ethics committee of the Samsung Medical Center Institutional Review Board. Patient records were anonymized prior to analysis.

For this retrospective study, we searched the electronic medical records of patients with submacular hemorrhage involving the fovea and who were treated with pneumatic displacement from March 2010 to September 2014 at Samsung Medical Center. During this period, submacular hemorrhages involving the foveal center that measured more than two disc areas were treated with pneumatic displacement. Submacular hemorrhages smaller than two disc areas, sparing the fovea, or that appeared chronic as evidenced by yellowish discoloration, signs of organized hemorrhage, and with a symptom duration longer than 2 months were not treated with pneumatic displacement. Eyes with a history of vitreoretinal surgery, presence of a macroaneurysm responsible for the submacular hemorrhage, proliferative diabetic retinopathy, or significant media opacity were excluded. The medical records of 49 patients who underwent intravitreal gas injection to displace a submacular hemorrhage related to exudative AMD were identified. We excluded 6 eyes that underwent consecutive vitrectomy due to dispersed vitreous hemorrhage after pneumatic displacement. An additional 6 eyes for which the follow-up period was shorter than 6 months were also excluded. Ultimately, 37 eyes were included.

Each patient initially underwent a comprehensive ophthalmic examination, including best-corrected visual acuity (BCVA) measurement, slit-lamp biomicroscopy, dilated fundus examination, fundus photography (IX50; Topcon, Paramus, New Jersey, USA), and OCT (Spectralis; Heidelberg Engineering, Germany, or Stratus; Carl Zeiss Meditec, CA, USA). For pneumatic displacement, a 0.3-ml volume of pure gas (perfluoropropane or sulfur hexafluoride) was injected intravitreally. Regarding the selection of gas, perfluoropropane gas was selected on the basis of physician’s discretion if intense buoyancy was required for displacement of hemorrhage. That is, we preferred to use pure perfluoropropane gas if the patient showed lower BCVA, thicker SMH, and loss of outer retinal integrity on OCT scan. At 6 hours after the procedure, the patients were instructed to maintain a face down position for 48 hours. The follow-up evaluation included BCVA, slit lamp biomicroscopy, funduscopy, and OCT. In all eyes, fluorescein angiography and indocyanine green angiography were performed using Spectralis HRA + OCT (HRA-2; Heidelberg Engineering, Germany) within 1 month after pneumatic displacement.

Demographic information, including age, gender, presence of hypertension and diabetes, and symptom duration, were collected.

Submacular hemorrhage area was measured using the intrinsic OCT software (Topcon IMAGENet Professional R-3.11). Submacular hemorrhages outside of 55° of the fundus photographs were not counted in the size measurement. The shortest radius of the submacular hemorrhage centered on the foveal center was measured, marking both the foveal center and the margin of the submacular hemorrhage using the intrinsic software.

### Optical coherence tomographic characteristics

The thicknesses of the neurosensory retina and submacular hemorrhage at the foveal center were measured using the manual caliper in the intrinsic software of the OCT. The presence of any defect in the external limiting membrane or in the ellipsoid zone was also evaluated.

**Fig 1:**
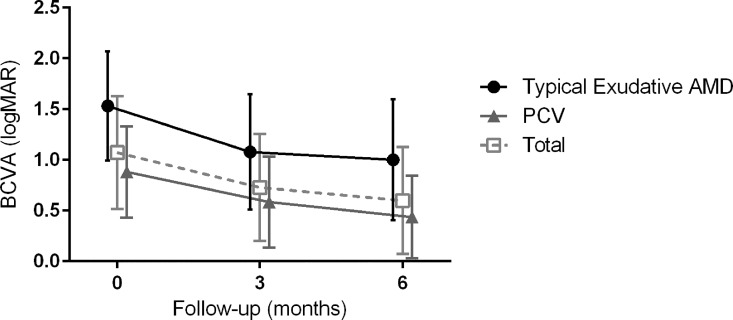
Change in best-corrected visual acuity (BCVA) after pneumatic displacement of the submacular hemorrhage. The mean BCVA improved significantly from baseline at 3 months (*P* < 0.001) and 6 months (*P* < 0.001). Patients with polypoidal vasculopathy showed a better BCVA at baseline (*P* = 0.002), 3 months (*P* = 0.015), and 6 months (*P* = 0.012) after treatment; however, the BCVA improvements were not different.

The reflectance of the submacular hemorrhage on the OCT image was quantified as an indirect measure of liquidity. For this measurement, OCT images obtained using the conventional, automated real-time mode with averaging over 90 frames were exported to a personal computer-based image analysis software package, Image J^®^ (version 1.45s, Wayne Rasband, National Institutes of Health, USA). Two independent examiners (G.E.C. and J.M.Y.) drew a 20-μm-diameter circle at the top of the subfoveal hemorrhage beneath the ellipsoid zone and quantified the grey scale of the circle (Fig 1). The mean value of the two measurements was regarded as the reflectance of the submacular hemorrhage.

### Treatment protocols after pneumatic displacement

The following treatment protocols after pneumatic displacement were administered as standard care for the patients.

1. Ranibizumab or bevacizumab was administered intravitreally if subretinal or intraretinal fluid was observed in the eyes with a typical pattern of exudative AMD or PCV without polyp(s). The eye was retreated with intravitreal injection of anti-VEGF at 1 month after the previous treatment if one of the following was observed: new or persistent fluid in the macula on OCT, increase of at least 100 μm in central subfield thickness according to OCT, or new submacular hemorrhage.
2. Standard-fluence photodynamic therapy was performed if active vascular polyp(s) was observed less than 1800 μm from the foveal center. The extent of irradiation included the polyp(s) and branching vascular network.
3. Focal laser photocoagulation was applied to ablate active vascular polyp(s) present more than 1800 μm from the foveal center. Angiographic evaluation was performed again if new or persistent fluid in the macula was evident on OCT or if an increase in foveal thickness of at least 100 μm according to OCT was detected within 3 months after each treatment. Further treatment guidelines after angiographic reevaluation were the same as described above.

### Statistical analyses

The decimal BCVA was converted to a logarithm of the minimal angle of resolution (logMAR) value. The change in BCVA from baseline was analyzed using the paired t-test. Fisher’s exact test was used for categorical variables, and the Wilcoxon rank sum test and independent sample t-test were used for comparison of continuous variables. Appropriate parametric analyses were performed when the data were normal. Among the possible prognostic factors, continuous variables except for the reflectance of submacular hemorrhage were evaluated using Pearson’s correlation analysis. The reflectance was analyzed using the Spearman correlation analysis. Stepwise regression analysis was used for determining predictive factors of visual prognosis. Statistical analysis was executed using SAS version 9.4 (SAS Institute, Cary, NC). A *P* value less than 0.05 was considered significant.

## Results

### Patient characteristics

This study included 37 eyes of 37 patients who underwent pneumatic displacement for treatment of submacular hemorrhage. (Datasets are included in S1 Appendix). There were 27 eyes with PCV and 10 eyes with typical exudative AMD. The mean age of the patients was 71.2 ± 8.2 years (mean ± standard deviation), and 21 patients (56.8%) were men. Patients with PCV were younger (*P* = 0.007) and more likely to be male (*P* = 0.001) compared to patients with typical exudative AMD. Of the 37 patients, 13 (35.1%) were treatment-naïve and were newly diagnosed with submacular hemorrhage. In the other 24 patients (64.9%), submacular hemorrhage developed during follow-up for previously diagnosed PCV (17 patients) or typical exudative AMD (7 patients). The baseline characteristics of the patients are summarized in Table 1. The results of an additional analysis of baseline characteristics according to subgroups of duration of symptoms (lasting at maximum one month vs. the others), selection of gas (perfluoropropane vs. sulfur hexafluoride), and pre-treatment (treatment-naïve vs. pre-treated) are presented as S1 Table.

**Table 1:** Baseline characteristics of 37 patients with submacular hemorrhage treated with pneumatic displacement, and the correlations between baseline characteristics and logMAR best-corrected visual acuity measured at 6 months.

	Mean ± SD	Median	Correlation coefficient	*P*-value
**Characteristics (*n* = 37 eyes)**				
Age, years	71.2 ± 8.2	72	0.482	0.003*
Male/Female, n (%)	21 (56.8)/16 (43.2)		-	0.015^†^
PCV/Exudative AMD, n (%)	27 (73.0)/10 (27.0)		-	0.012^†^
Diabetes, n (%)	8 (21.6)		-	0.482^†^
Hypertension, n (%)	21 (56.8)		-	0.741^†^
Anticoagulant, n (%)	12 (32.4)		-	0.438^†^
Baseline BCVA	1.08 ± 0.55	1	0.605	<0.001*
Symptom duration, days	16.1 ± 12.5	14	0.428	0.008*
Disease duration, months	20.8 ± 24.6	15	0.051	0.763*
Size of SMH, disc area	12.9 ± 8.3	10.57	0.139	0.412*
**Baseline OCT characteristics (*n* = 28 eyes)**				
Macular thickness, μm	122.7 ± 49.7	115.5	-0.218	0.266*
SMH thickness, μm	234.4 ± 152.5	211.5	-0.081	0.681*
Reflectance, AU (*n* = 21)	161.7 ± 34.4	155.88	0.473	0.030^‡^
SMH radius^§^, μm	1803.9 ± 891.9	1618	0.329	0.045*
Defect in ellipsoid zone, n (%)	11 (39.3)		-	0.016^†^
Defect of ELM, n (%)	9 (32.1)		-	<0.001^†^

logMAR: logarithm of the minimal angle of resolution; PCV = polypoidal choroidal vasculopathy; AMD = age-related macular degeneration; SMH = submacular hemorrhage; AU = arbitrary unit; OCT = optical coherence tomography; ELM = external limiting membrane

* Pearson correlation analysis

† Fisher’s exact test

‡ Spearman correlation analysis

§ The shortest radius of the submacular hemorrhage centered on the fovea

Additional treatments were performed for 33 patients at 20.4 ± 25.5 days after intravitreal pure gas injection. Intravitreal anti-VEGF injection (18 patients, 48.6%), photodynamic therapy (12 patients, 32.4%), and focal laser photocoagulation (3 patients, 8.1%) were used as the first additional treatments. No additional treatment was performed during 6 months of follow-up for three PCV patients and one typical exudative AMD patient. The number of intravitreal anti-VEGF injections was not significantly different (*P* = 0.320) between eyes with typical exudative AMD (2.1 ± 1.1) and those with PCV (1.6 ± 1.7) during the 6 months. The mean number of application of photodynamic therapy and focal lasers was 0.6 ± 0.7 and 0.3 ± 0.6, respectively, for eyes with PCV.

### Changes in visual acuity at 6 months and prognostic factors

The mean BCVA was 1.08 ± 0.55 at baseline, 0.74 ± 0.57 at 3 months, and 0.63 ± 0.58 at 6 months. Compared with the baseline value, the mean BCVA significantly improved at 3 months (*P* < 0.001) and at 6 months (*P* < 0.001). The mean BCVA of PCV patients was significantly better than that of typical exudative AMD patients at baseline (0.89 ± 0.42 vs. 1.58 ± 0.53, *P* = 0.002) and at 3 (0.58 0078 0.46 vs. 1.17 ± 0.61, *P* = 0.015) and 6 months (0.45 ± 0.45 vs. 1.10 ± 0.67, *P* = 0.012) (Fig 1).

Older age (*P* = 0.003), female gender (*P* = 0.015), lower baseline BCVA (*P* < 0.001), and longer symptom duration (*P* = 0.008) were significantly associated with a worse BCVA at 6 months. The results of the correlation analysis between the baseline characteristics and BCVA at 6 months are shown in Table 1.

Stepwise regression analysis revealed that baseline BCVA (β = 0.513, *P* = 0.001), symptom duration (β = 0.364, *P* = 0.010), and age (β = 0.310, *P* = 0.031) were significant prognostic factors of BCVA at 6 months.

### Optical coherence tomographic characteristics and visual outcome

**Fig 2:**
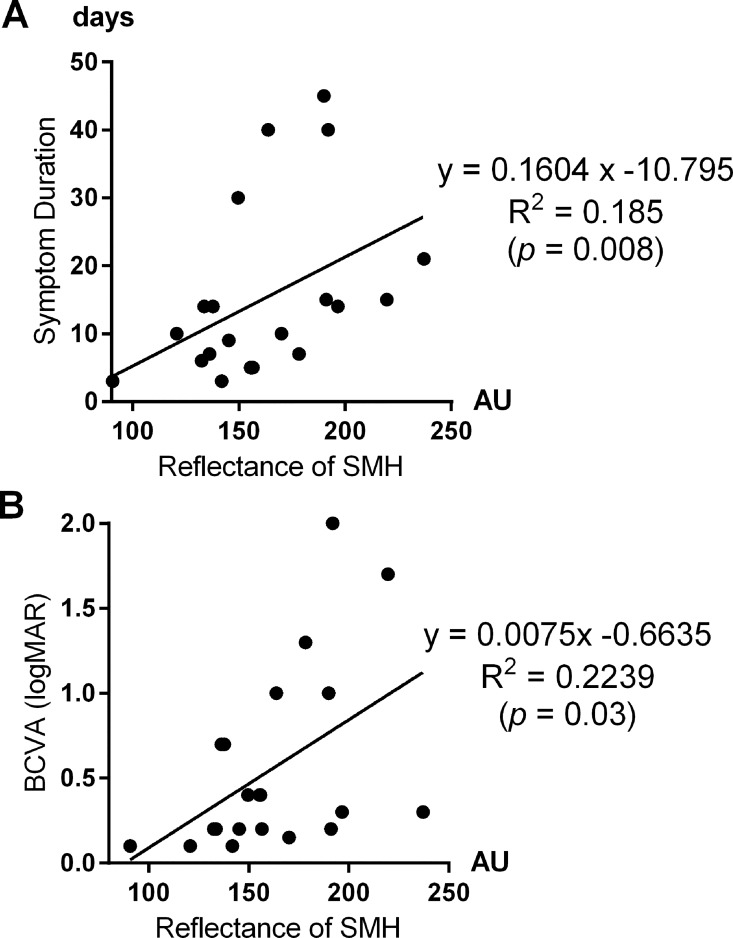
Reflectance of submacular hemorrhage (SMH) was positively correlated with symptom duration (2A, R^2^ = 0.185, *P* = 0.008). Reflectance was also positively correlated with logMAR best-corrected visual acuity (BCVA) at 6 months (2B, R^2^ = 0.2239, *P* = 0.030). In turn, high reflectance of the submacular hemorrhage on conventional optical coherence tomograph was associated with longer symptom duration and worse visual outcome at 6 months.

Submacular hemorrhage reflectance was quantified in 21 eyes, excluding eyes with a sub-RPE hemorrhage (*n* = 3) and those with only enhanced-depth-imaging mode OCT images (*n* = 4). The two measurement values obtained by two independent examiners were highly correlated (r = 0.875, *P* < 0.001). The mean reflectance was 161.7 ± 34.4 arbitrary units, and it was positively correlated with symptom duration (r = 0.510, *P* = 0.020). Reflectance was also positively correlated with logMAR BCVA at 6 months (r = 0.473, *P* = 0.030) (Fig 2).

That is, high reflectance of the submacular hemorrhage on conventional OCT was associated with longer symptom duration and worse visual outcome at 6 months. Among the OCT findings, the shortest radius of the submacular hemorrhage centered on the fovea (*P* = 0.045) and an intact ellipsoid zone (*P* = 0.016) and external limiting membrane (*P* < 0.001) were associated with better BCVA at 6 months. Among the OCT characteristics, PCV patients were more likely to have a shorter radius of submacular hemorrhage from the foveal center and an intact ellipsoid zone (*P* = 0.005) and external limiting membrane (*P* = 0.026) (Table 2). The correlations between the BCVA at 6 months and baseline characteristics including the correction of diagnosis variable (PCV *vs*. typical exudative AMD) are appended as S2 Table.

**Table 2:** Baseline characteristics of polypoidal choroidal vasculopathy and typical exudative age-related macular degeneration.

**Characteristics**	**PCV (*n* = 27 eyes)**	**Ex-AMD (*n* = 10 eyes)**	***P*-value**
Age, years	68.51 ± 6.0	78.6 ± 9.1	0.007^‡^
Male/Female, n (%)	20 (74.1)/7 (25.9)	1 (10.0)/9 (90.0)	0.001*
Diabetes, n (%)	6 (22.2)	2 (20.0)	1.000*
Hypertension, n (%)	17 (63.0)	4 (40.0)	0.274*
Anticoagulant, n (%)	10 (37.0)	2 (20.0)	0.224*
Disease duration, months	15.7 ± 16.5	22.5 ± 27.0	0.356^†^
Symptom duration, days	13.0 ± 11.3	24.4 ± 12.3	0.022^†^
Size of SMH, disc area	11.2 ± 6.8	17.5 ± 10.4	0.110^‡^
**OCT Characteristics**	**PCV (*n* = 23 eyes)**	**Ex-AMD (*n* = 5 eyes)**	***P*-value**
SMH thickness, μm	248.7 ± 141.3	168.4 ± 152.0	0.437^†^
Macular thickness, μm	128.1 ± 47.3	98.0 ± 58.7	0.331^‡^
SMH radius^#^, μm	1545.7 ± 636.2	2991.6 ± 998.35	0.029^‡^
Defect in ellipsoid zone, n (%)	6 (26.1)	5 (100.0)	0.005*
Defect of ELM, n (%)	5 (21.7)	4 (62.5)	0.026*

PCV = polypoidal choroidal vasculopathy; Ex-AMD = typical exudative age-related macular degeneration

SMH = submacular hemorrhage; ELM = external limiting membrane

* Fisher’s exact test

† Wilcoxon rank sum test

‡ Independent sample t-test

# The shortest radius of the submacular hemorrhage centered on the fovea

**Fig 3:**
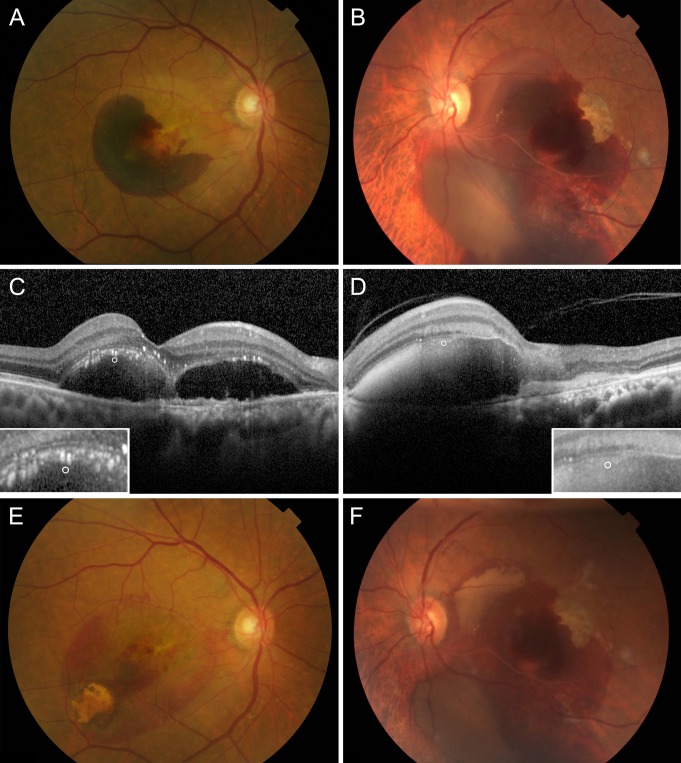
Fundus photograph and optical coherence tomograph of two distinctive cases: before and after pneumatic displacement of submacular hemorrhage. The shortest radius of submacular hemorrhage from the fovea was 393 μm for case 1 (A) and 1617 μm for case 2 (B). The insets of C and D show the magnified views of a 20-micron-diameter circle at the top of the hemorrhage, for which the grey scale was used for reflectance measurement. The reflectance (in arbitrary units) of cases 1 and 2 were 137.8 and 163.8, respectively (C, D). Two weeks after pneumatic displacement, submacular hemorrhage was significantly extruded in case 1 (E) but not in case 2 (F).

Representative cases in this study are demonstrated in Fig 3.

## Discussion

The current study demonstrated that pneumatic displacement and subsequent treatment such as anti-VEGF injection significantly improved visual outcome in patients with submacular hemorrhage secondary to PCV or exudative AMD. These findings reaffirm the results of previous studies demonstrating the efficacy of pneumatic displacement in patients with a thick submacular hemorrhage [4,7,8,10,12–15].

One strength of this study was the identification of OCT findings related to visual outcome in eyes with submacular hemorrhage. There was a significant positive correlation between lower submacular hemorrhage reflectance and improved final VA. Lower reflectance might represent greater liquidity of the hemorrhage, thus allowing it to be more easily displaced when treated with pneumatic displacement. Successful displacement can shorten both the time necessary for complete hemorrhage resorption and the exposure to toxic damage. Higher reflectance, in contrast, can be translated into organized or solid hemorrhage that is refractory to displacement. As far as the reporting physicians are aware, this is the first study to address the reflectance of a hemorrhage. Given the potential significance of reflectance, the development of intrinsic software in OCT for objective quantification of reflectance would be very useful.

In addition to reflectance, the shortest radius of the submacular hemorrhage centered on the fovea was significantly associated with visual outcome. If the shortest radius of the submacular hemorrhage centered on the fovea was short, it was more likely that the fovea was located at the periphery of the submacular hemorrhage. The peripheral portion of the hemorrhage overlying the fovea would be thinner compared to the portion located at the center, thus making it more likely to be completely displaced. Also, if the shortest radius is short, it can be expected that the causative vascular lesion is present in the extrafoveal area.

This study investigated baseline prognostic factors in exudative AMD with submacular hemorrhage initially treated with pneumatic displacement. Multiple logistic regression analysis revealed that better baseline BCVA, shorter symptom duration, and younger age were the significant prognostic factors of favorable visual outcome. Submacular hemorrhage with a long symptom duration has repeatedly been recognized as a poor prognostic factor [4,16]. The severity of outer retinal degeneration depends on the duration from initial bleeding to complete absorption of the hemorrhage because the metabolic supply of the outer retina depends primarily on the choriocapillaris. Also, a longer symptom duration indicates further clotting and, hence, less mobilization of the hemorrhage. This contributes to a greater delay in absorption of the hemorrhage despite pneumatic displacement and thereby contributes to poor visual outcome. It is worth mentioning that, because the onset of symptoms is frequently vague in elderly patients, the reflectance of the submacular hemorrhage, obtainable quantitatively using non-invasive method, can be more objective than a patient’s self-report of symptom duration.

We performed pneumatic displacement without tissue plasminogen activator injection, and the outcomes were comparable to those of previous studies involving the use of tissue plasminogen activator. Although encouraging results have been reported for a combination of tissue plasminogen activator injections with pneumatic displacement [4,7,14], subsequent comparison studies involving pneumatic displacement failed to reveal a significant difference in visual outcome between groups with or without tissue plasminogen activator injection [8,10,13]. Furthermore, the possible penetration of tissue plasminogen activator through the retina and the potential risk of complications, including rebleeding, vitreous hemorrhage, and retinal toxicity, were concerning [4,17–21]. Therefore, injection of tissue plasminogen activator should be reserved for selected cases of submacular hemorrhage such as those with longer symptom duration and high reflectance on OCT.

In the present study, PCV patients were more likely to have an intact ellipsoid zone and external limiting membrane, a smaller shortest radius of the submacular hemorrhage centered on the fovea, shorter symptom duration, better baseline BCVA, and younger age. These factors are likely related to the significantly better visual outcome in PCV patients, as has been noted in previous studies [22–25]. The current study focused on identifying the characteristics and prognosis of SMH itself and not the differences between the diseases. However, because typical exudative AMD and PCV might show different prognoses after pneumatic displacement of SMH, the etiologic diagnosis variables (PCV and typical exudative AMD) were adjusted in order to correct the possible bias.

This study had several limitations. The retrospective nature and the relatively small number of patients in this study might have limited the statistical power, especially for regression analysis. Also, applying two types of gases and not identical source of SMH could be potential sources of bias, although the final outcome showed no significant difference in this regard.

In conclusion, pneumatic displacement and follow-up treatment with anti-VEGF or photodynamic therapy resulted in a favorable visual outcome for patients with submacular hemorrhage related to exudative AMD. Among the baseline OCT characteristics, liquidity of the submacular hemorrhage, as represented by hemorrhage reflectance, the shortest radius of the submacular hemorrhage centered on the fovea, and the intactness of the ellipsoid zone and external limiting membrane were associated with visual outcome. These observations will help to select and counsel patients regarding pneumatic displacement as an initial treatment for submacular hemorrhage due to exudative AMD.

## Supporting Information

S1 Appendix Dataset of 37 patients who underwent pneumatic displacement for treatment of submacular hemorrhage. (XLSX)

S1 Table Baseline characteristics of 37 patients with submacular hemorrhage treated with pneumatic displacement, analyzed by the subgroups of duration of symptoms, selecton of gas, and pre-treatment. (DOCX)

S2 Table The correlations between baseline characteristics and logMAR best corrected visual acuity measured at 6 months including the correction of diagnosis variables (PCV and exudative AMD). The original results are described as uncorrected values.
(DOCX)
