# Supplementary material for: Optical Coherence Tomographic Features and Prognosis of Pneumatic Displacement for Submacular Hemorrhage
Source: PLoS One. 2016 Dec 19;11(12):e0168474. doi: 10.1371/journal.pone.0168474 (PMC5167395; doi:10.1371/journal.pone.0168474)
Supplement: S1 Table — (DOCX) [file pone.0168474.s002.docx]

**S1 Table. Baseline characteristics of 37 patients with submacular hemorrhage treated with pneumatic displacement, analyzed by the subgroups of duration of symptoms, selection of gas, and pre-treatment.**

|  | **Symptom Duration** | | | **Gas** | | | **Pre-treatment** | | |
| --- | --- | --- | --- | --- | --- | --- | --- | --- | --- |
|  | <1Mo n=27 | >1Mo n=10 | p-value | SF6 n=19 | C3F8 n=15 | p-value | No n=13 | Yes n=24 | p-value |
| **Characteristics (*n* = 37 eyes)** | | | | | | | | | |
| Age, years | 70.2 (72) | 74.1 (72.5) | .205 | 71.5 (72) | 71.3 (72) | .947 | 71.5 (72) | 71.1 (71) | .908 |
| Male/Female, n | 17/10 | 4/6 | .274 | 10/9 | 9/6 | .738 | 9/4 | 12/12 | .315 |
| Diagnosis |  |  | .037 |  |  | .560 |  |  | .766 |
| PCV | 4 | 6 |  | 4 | 5 |  | 3 | 7 |  |
| Exudative AMD | 23 | 4 |  | 15 | 10 |  | 10 | 17 |  |
| Diabetes, n | 6 | 2 | 1.000 | 3 | 4 | .672 | 2 | 6 | .685 |
| Hypertension, n | 16 | 5 | .717 | 12 | 8 | .728 | 8 | 13 | .739 |
| Anticoagulant, n | 9 | 2 | .688 | 6 | 5 | 1.000 | 5 | 6 | .465 |
| Baseline BCVA,  logMAR | 0.93 (0.8) | 1.47 (1.45) | .009 | 0.93 (0.8) | 1.22 (1.4) | .083 | 0.97 (0.8) | 1.13 (1) | .500 |
| BCVA at 6 months,  logMAR | 0.51 (0.3) | 0.94 (1) | .048 | 0.70 (0.4) | 0.63 (0.5) | .875 | 0.52 (0.3) | 0.68 (0.4) | .388 |
| Delta BCVA,  logMAR | 0.42 (0.4) | 0.53 (0.3) | .724 | 0.22 (0.3) | 0.59 (0.4) | .128 | 0.45 (0.4) | 0.45 (0.3) | .626 |
| Symptom duration,  days | 9.3 (9) | 34.5 (30.) | <.001 | 16.8 (10) | 16.2 (14) | .875 | 17.3 (10) | 15.5 (14) | .749 |
| Disease duration,  months | 22.2 (15) | 17.0 (4) | 1.000 | 24.4 (16) | 17.7 (1) | .401 | 2.8 (0) | 30.5 (26) | <.001 |
| Size of SMH,  disc area | 12.0 (9.2) | 15.2 (15.5) | .137 | 12.3 (8.4) | 13.4 (14.1) | .405 | 14.7 (16.5) | 11.9 (8.3) | .115 |
| **Baseline OCT characteristics (*n* = 21 eyes)** | | | | | | | | | |
| Macular thickness,  µm | 128.7 (118) | 100.5 (85) | .293 | 126.2 (118) | 119.7 (108) | .790 | 170.1 (170) | 104.6 (102) | .016 |
| SMH thickness,  µm | 203.4 (106.5) | 53.8 (46) | .165 | 110.5 (61.8) | 345.3 (232.3) | .064 | 168 (112.5) | 177.6 (56) | .414 |
| Reflectance, AU | 158.8 (155.5) | 173.9 (176.9) | .446 | 159.5 (155.7) | 167.0 (160.2) | .557 | 163.0 (154.5) | 161.2 (155.9) | .917 |
| SMH radius^§^, µm | 210.9 (211) | 249.8 (233.3) | .540 | 239.4 (213.5) | 170.3 (136.8) | .220 | 198.7 (151.3) | 226.1 (212) | .619 |
| Defect in ellipsoid zone, % | 35.3 | 50 | .618 | 14.3 | 83.3 | .007 | 50 | 33.3 | .631 |
| Defect of ELM, % | 25.5 | 25 | 1.000 | 21.4 | 33.3 | .613 | 16.7 | 26.7 | 1.000 |

Mean (median) values are presented.

SF6 = sulfur hexafluoride; C3F8 = perfluoropropane; PCV = polypoidal choroidal vasculopathy; AMD = age-related macular degeneration; BCVA = best corrected visual acuity; logMAR = logarithm of the minimal angle of resolution; SMH = submacular hemorrhage; AU = arbitrary unit; OCT = optical coherence tomography; ELM = external limiting membrane
