# Supplementary material for: Optical Coherence Tomographic Features and Prognosis of Pneumatic Displacement for Submacular Hemorrhage
Source: PLoS One. 2016 Dec 19;11(12):e0168474. doi: 10.1371/journal.pone.0168474 (PMC5167395; doi:10.1371/journal.pone.0168474)
Supplement: S2 Table — The original results are described as uncorrected values. (DOCX) [file pone.0168474.s003.docx]

**S2 Table. The correlations between baseline characteristics and logMAR best-corrected visual acuity measured at 6 months including the correction of diagnosis variables (PCV and exudative AMD).** The original results are described as uncorrected values.

|  | **Uncorrected**  **univariate**  ***P*-value** | **Uncorrected**  **multivariate**  ***P*-value** | **Corrected**  **univariate**  ***P*-Value** |
| --- | --- | --- | --- |
| **Characteristics (*n* = 21 eyes)** |  |  |  |
| Age, years | 0.003^*^ | 0.031 | 0.322 |
| Male/Female, n (%) | 0.015 ^*^ |  | 0.206 |
| PCV/Exudative AMD, n (%) | 0.012^†^ |  |  |
| Diabetes, n (%) | 0.482^†^ |  | 0.017 |
| Hypertension, n (%) | 0.741^†^ |  | 0.319 |
| Anticoagulant, n (%) | 0.438^†^ |  | 0.715 |
| Baseline BCVA | <0.001^*^ | 0.001 | 0.098 |
| Symptom duration, days | 0.008^*^ | 0.010 | 0.012 |
| Disease duration, months | 0.763^*^ |  | 0.909 |
| Size of SMH, disc area | 0.412^*^ |  | 0.536 |
| **Baseline OCT characteristics (*n* = 21 eyes)** | |  |  |
| Macular thickness, µm | 0.266^*^ |  | 0.407 |
| SMH thickness, µm | 0.681^*^ |  | 0.978 |
| Reflectance, AU (*n* = 21) | 0.030^‡^ |  | 0.046 |
| SMH radius^§^, µm | 0.045^*^ |  | 0.513 |
| Defect in ellipsoid zone, n (%) | 0.016^†^ |  | 0.589 |
| Defect of ELM, n (%) | <0.001^†^ |  | 0.393 |

PCV = polypoidal choroidal vasculopathy; AMD = age-related macular degeneration; BCVA = best corrected visual acuity; SMH = submacular hemorrhage; AU = arbitrary unit; OCT = optical coherence tomography; ELM = external limiting membrane
